# Decrypting the Potential of Lipidic Vesicular System for Delivery Enhancement of Tranexamic Acid in Melasma Hyperpigmentation Treatment

**DOI:** 10.34172/apb.025.45384

**Published:** 2025-09-01

**Authors:** Xin Lu Soo, Kang Nien How, Zee Wei Lai

**Affiliations:** ^1^School of Biosciences, Taylor’s University Lakeside Campus, 47500 Subang Jaya, Malaysia; ^2^Dermatology Unit, Department of Medicine, Faculty of Medicine and Health Sciences, Universiti Putra Malaysia, Serdang 43400, Malaysia; ^3^Dermatology Unit, Hospital Sultan Abdul Aziz Shah, Universiti Putra Malaysia, Serdang 43400, Malaysia

**Keywords:** Drug delivery, Hyperpigmentation, Lipidic vesicular, Melasma, Nanotechnology, Skin barrier, Tranexamic acid

## Abstract

Melasma is a prevalent pigmentary disorder characterized by irregular brown patches on sun-exposed face and neck regions, driven by increased vascular proliferation and dysregulated melanogenesis. Although benign, untreated melasma significantly impacts quality of life from emotional stress and cosmetic impairment especially for Asian women. Melasma complex and diverse aetiology involves melanocyte hyperactivity triggered by UVR exposure, genetics, hormones and aging. The effectiveness of current topical and physical therapies such as depigmenting agents, peels, photoablation and dermabrasion etc. have varying efficacy but limited by high recurrence rates. Tranexamic acid (TA) is a lysine-derived antifibrinolytic drug which has demonstrated high potential in reduction of melanogenic factors, inhibiting melanogenesis. Lipidic vesicular delivery systems including liposomes, ethosomes, niosomes, transferosomes and phytosomes showed extensive capability in the delivery of TA into deeper epidermal layers with improved stability and penetration efficacy. Multiple studies have shown that lipidic vesicular formulations of TA offer improved safety and efficacy compared to conventional delivery methods. However, further research and clinical trials will be necessary to verify the long-term safety and feasibility and to set up standardized protocols for this novel delivery system. Therefore, this review aims to scrutinize the potential of lipidic vesicles as a cutting-edge novel approach for the enhancement of TA’s efficacy in melasma hyperpigmentation treatment, as well as offering possibilities for future research and clinical applications in dermatology.

## Introduction

 Skin hyperpigmentation disorders particularly melasma is a highly prevalent pigmentary condition characterized by enhanced vascularization and disrupted melanogenesis in human skin. The most frequent melasma’s characteristic is the irregular appearance of contrast brown patches on the face or neck especially at sites of frequent solar exposure.^1^ Although melasma is medically benign, untreated cases can significantly impair life quality due to cosmetic concerns and associated psychological distress.^2^ Melasma affects all ethnicities with Asian women of reproductive age and darker skin phototypes accounting for up to 90% of cases.^3,4^ This condition develops when hyperactive melanocytes increase melanin synthesis and deposit excess pigment in the skin epidermis. Nevertheless, its pathogenesis is multifactorial including ultraviolet radiation (UVR), genetic predisposition, hormonal influences and aging.^5,6^

 Current melasma therapeutic approaches include topical depigmenting agents and procedural interventions but remain limited by high relapse and recurrence rates.^7,8^ Hydroquinone and retinoids are the most frequently prescribed topical depigmenting agents. The “New Trio” therapy cream containing isobutyl amide-thiazolyl-resorcinol, retinoic acid and dexamethasone have demonstrated comparable efficacy and tolerability to the gold-standard “Kligman’s Trio” comprising hydroquinone, tretinoin and corticosteroid in reducing Melasma Area and Severity Index (MASI) score by approximately 50% after 8-12 weeks of use.^9,10^ Furthermore, procedural treatments including laser therapy, microneedling, dermabrasion and acid-based chemical peels serve as secondary options. Combination treatments such as hydroquinone with laser therapy have shown more durable melanogenesis suppression than monotherapy.^8,11^ However, treatment effects remain inconsistent and adverse effects including post-inflammatory hyperpigmentation, erythema and barrier disruption compromise overall success. As a result, the pursuit for a safer, more effective and innovative therapeutic strategy for melasma remains an active area of research.

 Tranexamic acid (TA) has recently gained recognition as a promising therapeutic agent for melasma. Its’ first documented dermatological application was in 1979 by Sadako from Japan who observed a significant reduction in melasma severity within 2 weeks.^12^ TA is a synthetic lysine analogue with antifibrinolytic activity that hinders paracrine melanogenic mediators which induce melanocytes synthesis and has historically been used to prevent haemorrhagic episodes.^13^ Furthermore, TA competitively binds to the lysine-site of plasminogen, thereby inhibiting its conversion to plasmin and stabilizing the fibrin matrix. Additionally, TA directly inhibits tyrosinase activity thereby downregulating melanogenesis and reducing hyperpigmentation.^14^ Researchers have studied TA in topical, oral, intradermal and microneedle-assisted formulations reporting measurable reductions in pigmentation indices and improvements in patient satisfaction scores as a skin-lightening agent. Meta-analyses confirm that oral TA effectively reduces melasma pigmentation and is additionally beneficial in other skin diseases like eczema.^15-19^ Oral TA increases cutaneous vascularity and mast-cell density while attenuating epidermal hyperpigmentation.^18,20^ However, extensive oral administration may lead to multiple side effects like headaches, menstrual irregularities, gastrointestinal disturbances and rare thromboembolic events.^21-23^ On the other hand, OTC topical TA gels, creams and solutions have demonstrated favourable safety profiles.^24,25^ Multiple studies reported that topical TA yields moderate to marked pigment reduction with lesser systemic adverse effects compared with oral therapy.^26,27^

 Nevertheless, topical TA suffers from poor epidermal penetration and retention which severely compromises therapeutic efficacy.^16,28^ This is mainly due to its hydrophilic and zwitterionic structure causes repulsion by the lipophilic stratum corneum (SC) barrier, preventing adequate delivery to melanocyte-rich basal epidermis. Moreover, rapid systemic absorption reduces epidermal residence time which further attenuate its melanogenic impact.^29-31^ Consequently, topical TA treatment durations exceeding 3 months are often required to achieve visible improvement with recurrence frequently observed.^18^ To overcome these limitations, several methods especially the nanostructured delivery systems including lipidic vesicular system and polymeric based have been explored to enhance TA skin penetration.^32^ Among these, lipidic vesicular systems are especially promising due to their unique physicochemical features where their bilayer structure simultaneously solubilizes hydrophilic drugs and fuses with SC lipids, thereby improving drug deposition while maintaining safety. This review article therefore focused on exploring the novel concept of lipidic vesicular delivery system as a rational solution to the delivery limitations of topical TA, improving its efficacy and safety for melasma management.

## Melasma and hyperpigmentation

###  Overview of melasma and hyperpigmentation

**Figure 1:**
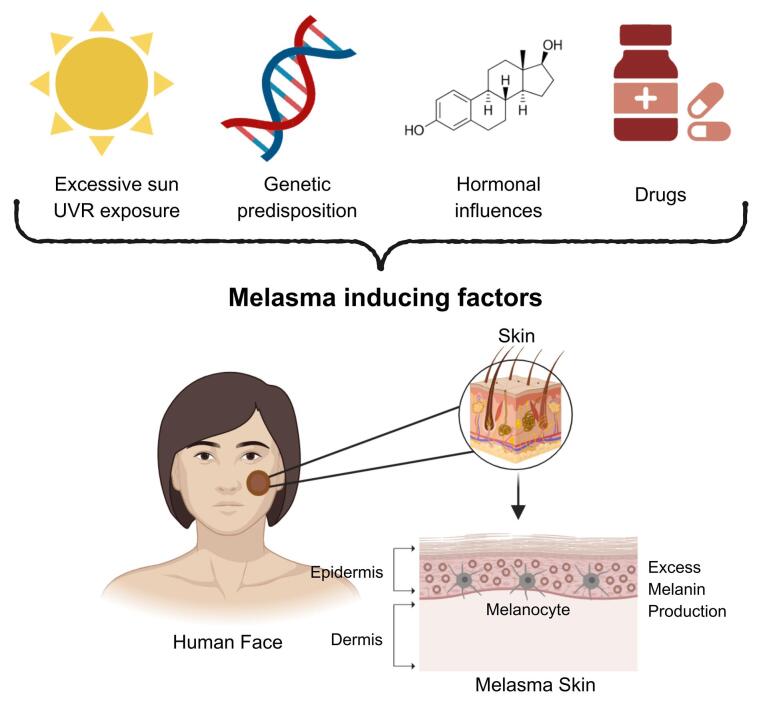
Key contributing factors of melasma: excessive sun UVR exposure, genetic predisposition, hormonal influences and drugs

 Melanogenesis is a complex biological process occurring within the melanocytes where melanin, the pigment responsible for photoprotection and integumentary coloration is produced. However, excessive melanin production by hyperactive melanocytes can lead to pigmentation disorders including melasma. Melasma is a common cosmetic ailment that can be identified by symmetrically distributed brownish facial patches and décolletage which worsen with extreme sun exposure.^3,6^ It has been categorized into three histologic variants: epidermal, dermal, and mixed which is distinguishable through Wood’s lamp examination and visible light assessment. The epidermal type is marked by increased melanin deposition across the epidermis, accompanied by enlarged melanocytes and a higher density of melanosomes. The dermal type is defined by the presence of melanophages in both the superficial and deep dermis whereas the mixed type exhibits histopathological characteristics of both epidermal and dermal patterns.^16,28^ The Melasma Area and Severity Index (MASI) score is the commonly used and validated tool for evaluating melasma severity and therapeutic outcomes.^33^ Although the precise pathogenesis remains unclear, multiple factors have been identified to aggravate melasma onset including UVR, genetic predisposition, hormonal dysregulation and medications shown in Figure 1.^34^

###  Pathogenesis of melasma and hyperpigmentation

**Figure 2:**
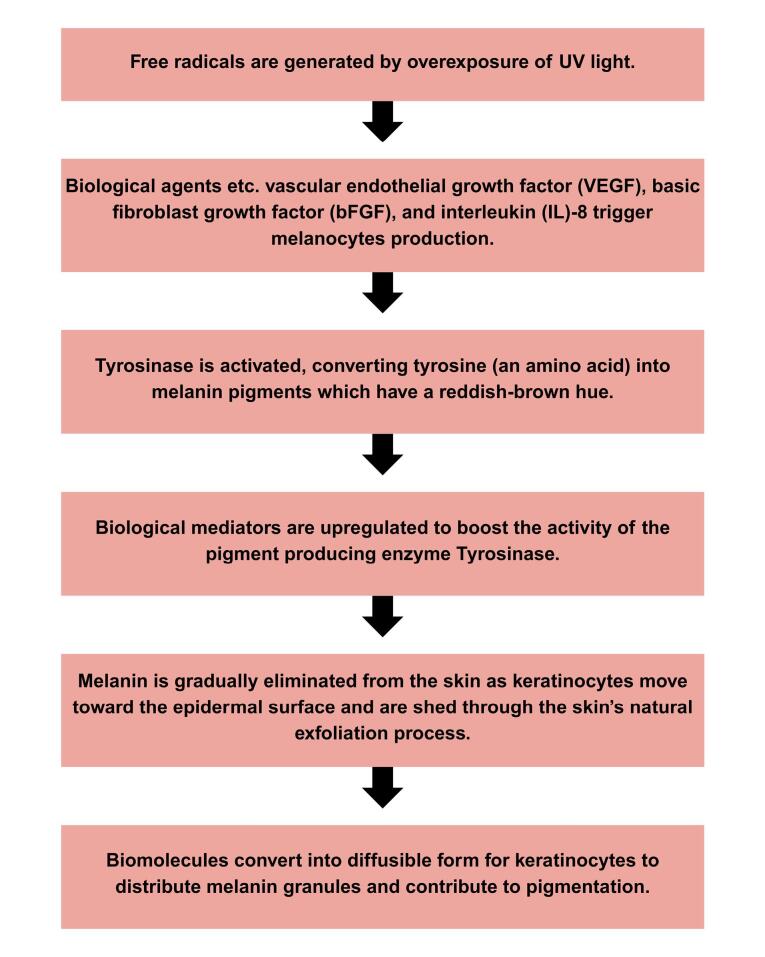
UV-induced pathway of melanin biosynthesis

 Numerous pathological explanations have been made in which UVR from sun exposure being the primary inducer of melanogenesis and melanosome transfer, triggering tyrosinase activity and leading to pigmentation as well as contributing to skin photoaging. Recent studies have confirmed the strong association between UVR and melasma development. Alcantara et al^35^ observed increased epidermal melanogenic response in both melasma-affected and adjacent normal skin after UVR exposure, regardless of extended exposure with photoprotection or brief uncontrolled exposure. Similarly, Sarkar et al^36^ reported that more than 50% of male melasma patients in their study were outdoor labourers and nearly 30% resided in mountainous regions with high sun exposure. These findings establish UVR as the major contributing factor to melasma development as it selectively darkens affected areas more than normal skin. UVR can activate melanocytes by inducing keratinocytes, fibroblasts and endothelial cells to secrete paracrine mediators that upregulate melanogenesis.^5,32,37^ The melanogenesis mechanism triggered by UVR is illustrated in Figure 2.

 Additionally, melasma involves dysregulation of more than 150 genes associated with both immediate and long-term modulation of pigmentation pathways. An individual’s inherent melanocyte count which governs melanin synthesis and skin colour is primarily determined by their genetic makeup.^38^ Significant ethnic variability in melasma prevalence has been observed, with familial clustering further implicating genetic predisposition as a major risk factor.^39^ These findings were in accordance with a multinational study by Ortonne et al^40^ involving 324 women where they reported 48% of the subjects exhibited a family history of melasma with 97% restricted to immediate relatives. Similarly, Tamega et al^41^ reported a familial history in 56% of 302 Brazilian patients involving identical twin sisters’ cases, supporting the genetic foundation of the condition. Facial melasma has been hypothesized to be inherited in a dominant fashion with environmental factors triggering onset in genetically susceptible individuals.^42^ Furthermore, darker skin individuals tend to have higher level of basal melanin and larger melanosomes which may further increase susceptibility.^43^

 Hormonal factors also contribute in melasma pathophysiology particularly oestrogen suggesting an increased risk in pregnant women, post-pubertal females and oral contraceptive users.^44^ Epidemiological studies suggest wide variability in hormone-related melasma prevalence across populations. For instance, Tamega et al^41^ reported that 36.4% of subjects in a Brazilian cohort experienced melasma onset during pregnancy, while 16.2% was caused by oral contraceptive use. Similarly, a cross-sectional study conducted in Indonesia revealed a melasma prevalence of 91.7% among 36 women taking oral contraceptives, demonstrating a positive correlation between incidence and usage duration.^45^ Elevated expression of oestrogen receptors (ER) and progesterone receptors (PR) has been consistently observed in melasma-affected dermal tissues at the molecular level.^46-48^ Upon binding to ERs, oestrogen activates rate-limiting tyrosinase in melanogenesis, thereby enhancing synthesis of melanin. Moreover, the upregulation of ion-exchange regulatory protein PDZ domain-containing kidney 1 (PDZK1) in melasma lesions has been found to potentially facilitating interactions between oestrogen signalling and ion transport mechanisms that promote melanogenesis and melanosome transfer.^39,49,50^ It was also postulated that oestrogen contributed to keratinocyte growth factor (KGF) production which affects melanocyte proliferation, tyrosinase activity and melanosome transfer ultimately resulting in melasma.^34,51^

## Tranexamic acid (TA)

###  Overview of TA

 TA is a haemostatic agent conventionally used since 1979 for the clinical management of irregular bleeding then repurposed for skin hyperpigmentation treatment.^12,52^ As a promising depigmentation agent, TA offers a viable topical treatment option for melasma individuals across varying degrees of severity. TA exhibits superior physicochemical stability with an oxidation half-life exceeding 24 hours at 40 °C as compared to other depigmentation agents such as arbutin, ascorbic acid, azelaic acid and glycolic acid which display variable stability and inconsistent success rates in melasma management.^53-54^ In a randomized controlled trial by Atefi et al,^26^ topical TA demonstrated non-inferiority to 5% hydroquinone achieving a mMASI score reduction to 2.30 as compared with HQ (P = 0.850) with a similar result reported by Sandeep et al^55^ Furthermore, direct comparisons with arbutin which is another standard agent remain limited but available data suggest TA achieves numerically greater MASI reductions (6.2 vs 3.4) without reaching statistical significance in small cohorts. Mechanistically, TA functions as a plasmin inhibitor thereby reducing the production of inflammatory mediators essential for melanogenesis.^56^ It can be administered via topical (2%-5%), intravenous intradermal (4 mg/mL) and oral dosing (500-1000 mg daily) with combination therapy often employed to enhance depigmentation efficacy.^49^ However, long term oral administration specifically more than 6 months is associated with a 2-4 % incidence of gastrointestinal disturbance and < 0.1% absolute risk of thromboembolic events. In contrast, topical or intradermal TA is associated with mild, transient erythema or irritation ( < 15% incidence) and no reported vascular events.^18,21^

###  Mechanism of TA in melasma hyperpigmentation

 The precise mechanism by which TA attenuates melasma hyperpigmentation remains under investigation. Limited studies suggest that TA primarily acts through suppression of plasmin activity, thereby disrupting melanocyte activation and melanin synthesis. TA inhibits plasminogen’s lysine-binding sites, effectively suppressing its conversion to plasmin and subsequently downregulating melanocyte-keratinocyte interactions that drive melanogenesis.^57^ Maeda and Tomita^58^ demonstrated that TA impedes melanocytes’ melanin synthesis by blocking the plasminogen-plasmin pathway and preventing melanocyte-keratinocyte interaction. Similarly, Maeda and Naganuma^59^ reported that TA reduced melanocyte tyrosinase activity in UVR-induced hyperpigmentation guinea pigs by preventing plasminogen binding to keratinocytes. Additionally, TA may suppress melanogenesis by inhibiting plasminogen-keratinocyte interaction which reduce prostaglandin and free arachidonic acid synthesis as well as tyrosinase activity. These findings were supported by a split-face study involving 40 melasma patients.^60^ Renckens et al^61^ suggested that TA inhibits plasmin activity on fibrin and cells by competitively interacting to plasminogen’s high-affinity lysine sites. Thus, it prevents the surface-mediated conversion of plasminogen to plasmin.

 Beyond melanocyte regulation, plasmin is also crucial in angiogenesis. Plasmin-mediated degradation of extracellular matrix-bound vascular endothelial growth factor (VEGF) generates diffusible VEGF forms that promote neovascularization. As a plasmin inhibitor, TA ceases basic fibroblast growth factor (bFGF)-induced angiogenesis, thereby indirectly reducing pigmentation.^62-63^ Zhu et al^1^ reported that TA significantly inhibited tyrosinase activity, melanin synthesis and VEGF-induced melanogenic protein expression when VEGF receptors were neutralized. Similarly, in a clinical study involving 25 melasma women, topical TA demonstrated its ability to suppress bFGF and VEGF-mediated angiogenesis, resulting in decreased melanogenesis.^20^ Additionally, TA exhibits structural resemblance to tyrosine, enabling competitive inhibition of tyrosinase which further enhance its depigmentation efficacy.^14,15^ Although the exact molecular pathways remain incompletely defined, current evidence suggests that TA exerts its anti-melanogenic effects through two mechanisms: (i) plasmin inhibition which disrupts inflammatory and angiogenic pathways, and (ii) direct enzymatic inhibition of tyrosinase. Collectively, these mechanisms contribute to the clinical efficacy of TA in reducing melasma pigmentation across diverse patient populations.

###  Pharmacologic of TA for melasma hyperpigmentation treatment

 Pharmacological therapy of TA for melasma involves oral or topical administration. Oral administration remains the most conventional approach with multiple clinical trials confirming its efficacy in managing melasma-related hyperpigmentation. A placebo-controlled randomized study administering 250 mg of TA twice daily for 3 months to patients with moderate-to-severe melasma demonstrated significant improvements particularly in severe cases but effects were not sustained post-treatment.^18^ Furthermore, Bhattacharjee et al^64^ further compared TA administered at 250 mg and 500 mg two times per day, observing equivalent therapeutic outcomes with no notable differences in tolerability. Similarly, Wang et al^14^ assessed 250 mg TA administered twice versus thrice daily over 12 weeks, revealing comparable therapeutic outcomes. A prospective clinical trial examined oral TA dosages from 500 to 1500 mg/d over treatment periods from one month to two years, finding progressive MASI score reductions across all groups with no significant differences between doses.^65^ These findings indicate that oral TA is effective starting at 250 mg, with therapeutic effectiveness more dependent on duration than dosage. However, prolonged use may reduce adherence due to common side effects such as gastrointestinal damages and menstrual irregularities.^18,21-23^

 In addition to the oral administration route, TA has demonstrated skin-lightening effects when applied topically at concentrations between 2-5%.^20,32^ In a study by Yoo et al^27^ reported substantial melasma improvement over 10 weeks with a TA-containing cream, finding MASI score reduction and high patient satisfaction. Another trial showed that 2% TA cream reduced hyperpigmentation in mild melasma patients with no adverse effects observed after three months.^24^ According to Fox’s^25^ clinical study, 80% of subjects experienced significant melasma improvement after using a TA emulsion for 6 months with no adverse effects. Similarly, a comparative trial between 5% TA solution to a 2% hydroquinone (HQ) solution in 60 women with epidermal melasma found TA particularly effective. The TA group reported a 33.3% satisfaction rate which is greater than 6.7% observed from HQ group, attributed to faster visible effects and minimal side effects.^26^ These findings suggest that topical TA can effectively reduce melasma severity, although its onset of action may be slower than oral administration. Therefore, further research is required to optimize topical TA formulations for improved skin penetration and to accelerate visible clinical results.

## Novel lipidic vesicular approach in TA delivery

###  Overview of lipidic vesicular system 

**Figure 3:**
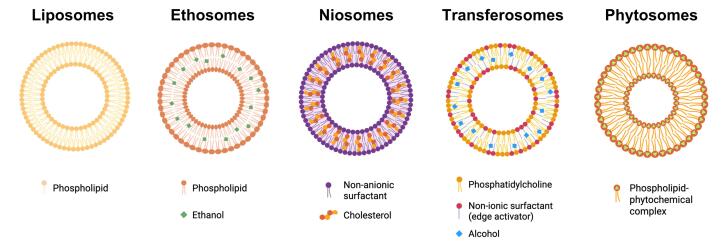
Schematic representation of lipidic vesicular systems including liposomes, ethosomes, niosomes, transferosomes and phytosomes

 Lipidic vesicular systems have gained significant attention in cell membrane biology emerging as a promising approach for trans-epidermal drug administration.^66^ Their ability to facilitate delivery of encapsulated drugs while acting as a depot system enhances stratum corneum (SC) penetration and facilitates continuous drug release by acting as a membrane barrier that controls skin absorption rates.^67-69^ Lipidic vesicles are organized spherical structures containing single or multiple concentric bilayers, formed through amphiphilic molecules’ self-assembly in water.^70^ Various types of lipidic vesicular drug delivery systems such as liposomes, ethosomes, niosomes, transfersomes and phytosomes have been developed for the delivery enhancement of depigmenting agents and TA to improve melasma hyperpigmentation treatment (Table 1). Figure 3 provides a schematic illustration of various vesicle structural organizations.

**Table 1:** Various lipidic vesicular carriers with their advantages and drawbacks

**Lipidic carriers**	**Characteristics**	**Advantages**	**Disadvantages**	**Ref.**
Liposomes	- Composed of phospholipids and cholesterol - Amphiphilic hollow lipid bilayer sphere	- Able to encapsulate both hydrophobic and hydrophilic drug molecules - Non-toxic and biodegradable	- Large particle size - Susceptible to oxidation and degradation - High manufacturing cost	^71-74^
Ethosomes	- Composed of 20-45% ethanol, phosphatidylcholine, cholesterol and water	- High elasticity - Superior skin penetration and systemic circulation	- May cause skin irritation if ethanol content exceeds 30% - Solubility and compatibility issues for certain drugs	^75-77^
Niosomes	- Single or multilamellar - Developed from non-ionic surfactants in combination with cholesterol	- More stable and lower formulation cost than liposomes - Non-ionic surfactants enhance vesicle stability	- Time consuming process for manufacturing - Limited long-term in vivo safety data - Potential surfactant toxicity	^66,78-80^
Transferosomes	- Ultra-flexible vesicle - Aqueous core encapsulated within a phospholipid bilayer with edge activator	- Highly elastic and deformable - Effective for large and hydrophilic molecules	- Difficulty in encapsulating hydrophobic drugs - Complex physicochemical optimization needed	^66-67,81-82^
Phytosomes	- Molecular complexes formed between phospholipids and polyphenols	- Cost-effective - Synergistic enhancement of bioactive compound delivery and therapeutic efficacy.	- Reduce concentration of active ingredient due to dependency on plant material - Limited clinical data for safety and efficacy	^83-84^

###  Liposomes

 Liposomes were initially used in membrane research in 1965 and were later proposed as drug delivery systems in 1972.^71^ Conventional liposomes primarily composed of phospholipids and cholesterol. Amphiphilic phospholipids with polar heads and nonpolar tails are the main building blocks of liposomes which enable them to encapsulate both water and lipid soluble substances.^72^ Consequently, liposomes represent a significant advancement in drug and cosmetic delivery by enhancing stability, possessing high biocompatibility and low toxicity. Empty liposomes exhibited 100% cell viability, while drug-loaded liposomes maintained over 50% cell viability following 24 hours exposure at a 5.37 µg/mL concentration in Vero cell line cultures derived from monkey kidney. The observed cytotoxicity was found to be concentration-dependent, with increasing drug concentrations corresponding to a higher percentage of cell growth inhibition.^85^ This finding aligns with Nguyen et al^86^ observations who suggested that increased cytotoxicity associated with liposomal formulations may result from enhanced cellular uptake.

 According to studies, liposomes also enable targeted drug delivery and improved pharmacokinetics.^66,87^ Liposomes enhance drug delivery by fusing with cell membranes and disrupting SC lipid organization, thereby facilitating transdermal penetration.^68,87^ Additionally, factors such as drug molecule size, oil-water distribution coefficient and lipid membranes interactions can influence delivery efficiency within liposomes.^72^ Studies by Kirjavainen et al^88^ and Maghraby, Williams, and Barry^89^ suggested that liposomes promote drug absorption by modifying the skin’s outermost layer, thereby enabling increased pharmaceutical uptake and enhance skin penetration. However, liposomes clinical application may be limited by high production costs with expenses exceeding over US$ 1000 per gram of active pharmaceutical ingredient and requiring cold-chain storage which further increases logistical complexity and cost.^73-74^

 Liposome have been widely utilized for various drugs notably for TA, where the presence of lipids within liposomes may reduce irritation and provide long-lasting moisturizing benefits.^90,91^ TA is incorporated within the aqueous phase of liposomes due to its hydrophilic nature. According to studies, it was hypothesized that liposomes enhanced TA percutaneous transport through merging the phospholipid bilayer with intercellular lipid to create transient hydrophilic channels that circumvent the intact SC and improved drug accumulation in follicular openings.^92,93^ Liposomes have demonstrated effective transdermal delivery of TA using various lipid compositions with diverse surface charges, including hydrogenated soya phosphatidylcholine, cholesterol, stearyl amine and dicetyl-phosphate.^94^ Moreover, TA-entrapped liposomes exhibit enhanced physical stability, high drug entrapment efficiency ( > 90% for up to two months) and smaller particle sizes, which enhance skin penetration and sustain drug release.^95^ However, these formulations exhibit increased leakage and degradation at elevated storage temperatures. This temperature-dependent instability is attributed to increased lipid fluidity which facilitates drug leakage, underscoring the importance of maintaining storage conditions at 4 °C to ensure product stability.

 In a 12 weeks split-face clinical study involving 30 women, Banihashemi et al^96^ evaluated 5% topical liposomal TA with no reported discomfort while yielding significant superior MASI scores reduction as compared to conventional treatments. These findings highlight its potential as a novel and safe therapeutic modality for melasma. Similarly, a study using 1.8% liposomal TA in 60 patients with melasma demonstrated over 50% improvement, further supporting its efficacy.^97^ Furthermore, Politranexamide® which is a patented liposomal TA emulsion achieved a significant MASI score reduction from 10.73 to 7.75 in facial melasma treatment.^98^ Choo and Tey^93^ conducted a comprehensive study evaluating the in vitro skin-lightening effects of TA encapsulated in poly(lactic-co-glycolic acid) (PLGA)-based polymeric nanoparticles and in liposomes. Liposomal-TA demonstrated superior efficacy, achieving greater reduction in melanin content despite containing only half the concentration of TA (0.25% vs 0.5%) as compared with PLGA-TA. This enhanced penetration may be attributed to the compositional similarity between liposomal phospholipids to skin membrane lipids, which facilitates permeation across the SC via both intracellular and transcellular pathways.^92^

###  Ethosomes

 Ethosomes are specialized lipidic vesicles containing phospholipids, water and 20-45% (v/v) ethanol, first reported by Touitou et al^75^ which markedly improve transdermal drug delivery.^76^ According to studies, the interaction between ethanol, vesicles and skin’s lipid bilayer significantly impact SC fluidity, enhances skin compatibility and drug penetration. Its mechanism of action involves ethanol-induced SC lipid fluidisation, dissolution of intercellular lipids and a downward shift in the SC-phase transition temperature thereby increasing membrane permeability, facilitating drug penetration and retention.^99-102^ Compared with liposomes (EE ≈ 60-70%), ethosomes exhibit 1.5-2 times higher entrapment efficiency (EE ≈ 85-95%) and 3-4 times greater deformability index, enabling 2-6 times deeper permeation into viable epidermis in ex-vivo Franz-cell studies utilising excised rat skin.^103^ They outperform conventional liposomes in exhibiting higher drug-entrapment efficiency and superior deformability, facilitating the efficient delivery of water and lipid soluble drugs through the SC into deeper viable layers of the skin.^75,104,105^ Consequently, ethosomes have been investigated for various skin conditions treatment including acne, psoriasis, melasma and atopic dermatitis, yet < 10% of these studies have progressed beyond ex-vivo human to controlled clinical trials.^106^

 Nonetheless, the presence of ethanol in ethosomes formulation possess several drawbacks including volatility, risk of cutaneous irritation and reduced stability which may negatively impact formulation quality and shelf life. In contrast, Mehmood et al^107^ reported that a vitamin D-loaded ethosomal gel did not produce any irritation on rabbit skin after 24 hours of application in their in vivo study. These findings suggest that ethosomes may be considered relatively safe and non-toxic for topical use. An increase in ethanol concentration has been shown to improve drug molecules diffusion across skin barrier with optimal effects observed at concentrations up to 30%. This enhancement is attributed to ethanol’s ability to fluidize lipid bilayers and increase membrane permeability. However, further increase of ethanol content beyond 30% specifically at 45% has been reported to inhibit the diffusion efficiency of the active compound due to excessive permeability of the ethosomal membrane.^77^ This destabilization of the ethosomes structure ultimately compromises the integrity and performance of the delivery system.^102^ Hence, modified ethosomal systems utilizing propylene-glycol, glycerol or phytoconstituent such as curcumin have been developed as ethanol substitutes with the purpose of mitigating these limitations. These alternatives provide better stability, lower volatility and enhanced sensitive skin compatibility.^108,109^

 A study by Niu et al^110^ demonstrated that drug-loaded ethosomes fusing with SC lipids and breaching the barrier to reach the viable epidermis with an EE of 78.21% and up to 95% of drug permeation in ex-vivo transdermal diffusion cell study in excised pig skin. Furthermore, Guo et al^29^ conducted a randomized double-blind clinical trial (n = 88, Fitzpatrick III-IV) investigating 0.5% TA-loaded ethosomes with 30% ethanol for melasma. Results showed that vascularization-related melasma significantly improved in the TA-loaded ethosomes group likely due to the elevated ethanol concentration which disrupts the SC enhancing drug delivery by passive transport.^111^ Shaji and Parab^112^ introduced transethosomes (ethosomes containing edge activators) that achieved 94% of EE and 93.97% in TA deposition within the viable epidermis in excised porcine abdomen skin showing enhanced flexibility and penetration capability. These optimized ethosomes offer a simple production process, improved scalability and a reduced TA dosage requirement, thereby minimizing toxicity risks and improving patient compliance.^113^ However, TA-loaded ethosomes have received limited research attention compared to other drugsand further clinical studies with objective biomarkers and irritation scoring are necessary to thoroughly evaluate their safety and effectiveness against melasma.^114-116^

###  Niosomes

 Niosomes are described as nanocarrier vesicles that are formed through the self-assembly of non-ionic surfactants with size distributions between 10 and 1000 nm. The surfactants most utilised in niosomes are polyoxyethylene alkyl ethers and sorbitan esters which are non-toxic, biodegradable, biocompatible and chemically stable.^78,79^ For instance, span60/cholesterol-based niosomes loaded with mangosteen extract for wound healing applications preserved ≥ 80% viability of murine fibroblast cells across all tested concentrations (10%-100%) and were classified as non-irritating in in vivo rabbit skin studies. The reported irritation index was 0.29, with no observable signs of erythema or oedema up to 72 hours post-application.^117^ Research on niosomes has substantially increased in recent years due to their nanoscale size and amphiphilic characteristics which permit the encapsulation of a wide range of pharmacological molecules and enhances cutaneous penetration.^118^ Niosomes were designed as chemically stable and economical liposomes substitutes, offering improved bilayer rigidity and achieving up to 90% entrapment efficiency with approximately 85% drug release.^119^ Unlike liposomes, niosomes avoid oxidative degradation, reduce raw material costs by 30%-50% and minimize variability by replacing oxidation-prone phospholipids with non-ionic surfactants.^66,80^ Furthermore, niosomes functions as intradermal drug depots, facilitating sustained drug release and enable targeted delivery while reducing dosage requirements.^120-123^ Its mechanism of action involves surfactants-induced SC lipid fusion and modification of thermodynamic activity gradient thereby promoting transdermal drug penetration.^68,124,125^

 Niosomes were first identified for cosmetic applications in 1972. However, peer-reviewed investigations specifically addressing TA-loaded niosomes remain limited with ≤ 10 studies indexed in PubMed/Web of Science as of July 2024, highlighting a critical knowledge gap that requires systematic exploration. The first commercial niosomal anti-aging cream was introduced by Lancôme in 1987, followed by L’Oréal’s patented “Niosôme^TM^” technology which further validated industrial-scale reproducibility with > 95 % batch uniformity.^126,127^ Given their unique physicochemical properties, niosomes have been widely studied as delivery systems for active compounds including cannabidiol, forskolin, caffeine and aescin, offering hydrating, anti-aging, antioxidant, anti-inflammatory and skin-whitening benefits.^124,128,129^ Their ability to improve chemical stability and enhance cutaneous absorption especially for poorly soluble drugs, makes them highly attractive for cosmetic and dermatological applications.^130,131^ In a study published in 2010, Shatalebi et al^132^ demonstrated that N-acetyl glucosamine-loaded niosomes (500-4500 nm) increased skin retention by 42% relative to control solution and maintained up to 24 hours of steady-state flux, indicating promising efficacy for hyperpigmentation treatment.

 Further studies have investigated the development of hydroquinone-loaded niosomal gel to enhance skin depigmentation. Hydroquinone is a commonly used depigmenting agent where excessive concentrations may cause skin irritation including burning and redness. Ammar et al^133^ formulated a Span-80/cholesterolniosomal topical gel with 98% of EE and achieving97% in vitro hydroquinone release. A randomized comparative clinical trial further demonstrated up to 85% improvement in therapeutic impact over 12 weeks treatment period. This trial also reported minimal to no adverse effects, which were attributed to the high encapsulation efficiency in niosomes that reduce hydroquinone direct contact on skin. Similar findings were also reported by Divanbeygikermani et al.^134^ Moreover, several other studies have also confirmed the efficacy of niosomes in cosmeceutical applications.^135,136^ Focusing on transdermal delivery, niosomes has outperformed both liposomes and ethosomes in terms of drug release kinetics and entrapment efficacy. This is primarily attributed to surfactants incorporation which stabilizes vesicle membrane and impart high deformability, hence enhancing penetration through SC bypass.^137^ Nevertheless, optimization of niosomes’ formulation specifically TA-loaded remain under-investigated.^138^ Systematic investigations are required to determine the optimal surfactant-to-cholesterol molar ratios, preparation methods and concentration of TA to facilitate clinical translation for hyperpigmentation and melasma treatment.

###  Transferosomes

 Transferosomes are recognized as a novel variant of liposomes distinguished by their superior deformability. They are primarily composed of phospholipids forming a bilayer membrane, an edge activator (10%-25%), ethanol ( < 10%), and water as the dispersion medium. These vesicles typically exhibit particle sizes under 300 nm, possess exceptional elasticity through the incorporation of edge activators that destabilize and alter the lipid bilayer. Commonly employed edge activators comprise non-ionic surfactants (Tweens, Spans) and bile salts (etc. sodium deoxycholate).^66^ Structurally, transferosomes consist of a phospholipid bilayer enclosed aqueous core where hydrophilic active agents are encapsulated in the core and hydrophobic compounds are solubilized within the membrane. The inclusion of edge activators reduces interfacial tension and alters bilayer assembly, making transferosomes highly malleable and capable of intercalating into the SC lipid matrix without compromising vesicle integrity or cargo retention.^67^ This extreme deformability enables transferosomes to bypass the SC barrier, where they gradually release their payload into deeper skin layers while simultaneously protecting the active compounds from metabolic degradation.^81^

 In comparison to conventional liposomes, transferosomes are not only non-toxic, biocompatible and biodegradable but also exhibit superior physical stability and enhanced transdermal penetration efficiency.^82^ In a study by Wadher et al,^139^ blank phospholipid/sodium-deoxycholate transferosomes exhibited no cytotoxic effects on normal human cell lines across concentrations ranging from 0-15 mg/mL. Given their superior deformability and non-cytotoxicity, transferosomes are particularly suitable for cosmeceutical applications, especially in the topical delivery of antioxidants. For instance, Li et al^140^ developed ascorbic palmitate (AP)-loaded for improved melasma treatment. Both in-vitro and in-vivo studies demonstrated a 14.1-fold increase in AP skin permeability and superior anti-melasma efficacy, with effective attenuation of oxidative stress and inflammation, and no observable signs of skin irritation. Similarly, Lee et al^141^ formulated niacinamide-loaded transferosomes which demonstrated markedly enhanced skin penetration and whitening efficacy relative to conventional liposomes, highlighting their potential in melasma and hyperpigmentation management. However, to date, the development of TA-loaded transferosomes remains largely unexplored. Owing to the high deformability of transferosomes, it is scientifically reasonable that TA-loaded transferosomes could bypass the SC and enhanced transdermal absorption efficacy,^82^ where further investigation is required.

###  Phytosomes 

 Phytosomes also known as phytophospholipid complexes, are advanced lipidic vesicular systems that integrate phospholipids with biologically active phytochemicals through bonding of hydrogen, typically between the hydrophilic regions of both molecules. This delivery technology was first introduced by Indena Company in the late 1980s to address poor oral bioavailability of certain plant-derived compounds that suffer from extensive first-pass metabolism and limited membrane permeability which restricts their therapeutic potential.^83^ Structurally, phytosomes resemble conventional liposomes but offering more advantages, including higher encapsulation efficiency, superior physicochemical stability and significantly enhanced absorption and bioavailability of active ingredients.^84^ Transdermal delivery of bioactive molecules via phytosomes can occur through various pathways across the SC including intercellular (sweat and sebaceous glands, hair follicles) and intracellular (lipid matrix and corneocytes) routes. It has been reported that drug encapsulation within phytosomes results in a lipid-soluble complex that can interact with both lipid and water-based environments thereby increasing the drug’s diffusion coefficient and enhancing its partitioning into the SC can significantly improve skin permeability.^142,143^

 Phytosomes are typically fabricated within a particle size range of 50 nm to several 100mm, allowing for formulation flexibility tailored to specific application requirements.^83^ Over the past decade, numerous botanical extracted phytochemicals have been successfully incorporated into phytosomes for both dermatological and cosmetic applications. For instance, Priani et al^144^ formulated a topical phytosome serum loaded with cocoa-pod extract which demonstrated high EE of 91% with a particle size of 672 nm. This formulation also exhibited strong antioxidant activity with an IC_50_ value of 199.98 ppm which is comparable to a commercial whitening product (Hadalabo Ultimate Whitening Milk). Similarly, Patel et al^145^ developed and optimized arbutin/phosphatidylcholine complexes to address the limited skin permeability of arbutin as a skin-whitening agent. The optimized formulation showed an enhanced in-vitro drug release profile as compared to aqueous formulation (84.8% vs 53.15%), indicating improved cutaneous absorption. In comparison to other vesicular carriers, phytosomes represent a unique class of bioactive delivery systems in which the phospholipids not only serve as a vehicle but also exhibit membrane-repair and anti-inflammatory benefits.^146^ Therefore, the integration of phytosomes with TA may present promising approach to improve skin permeability and bioavailability while potentially exerting synergistic effects through both active components.

## Limitations and Challenges

 Lipid-based vesicular drug delivery systems have attracted considerable attention in contemporary research, particularly in dermatology and cosmeceuticals. Nonetheless, the development of TA-loaded lipidic vesicles faces substantial challenges in formulation, regulation and clinical translation. From a formulation perspective, liposomal TA requires ≥ 98% of ultra-pure phospholipids and specialized techniques such as thin-film hydration, both of which significantly increase production costs.^147^ In addition, liposomes are highly susceptible to oxidation and degradation which compromises long-term stability. Ethosomes containing > 30% ethanol also exhibit reduced stability and drug leakage under elevated temperature or humidity, complicating storage and transportation. High ethanol concentrations may further induce skin irritation or erythema if not carefully optimized and can negatively impact drug solubility, stability and bioavailability.^148^ Other vesicular systems including transferosomes, niosomes and phytosomes present additional challenges related to complex physicochemical behaviour, excipient interactions and a lack of robust in vivo safety data. In particular, the edge activators and surfactants utilised which are essential for improving deformability and penetration may also pose long-term toxicity risks if not optimized.^149^

 Beyond formulation, regulatory gaps further complicate translation. Currently, no country has established legislation specifically dedicated to nano-systems in dermatology or cosmetics. For instance, neither the U.S. Food and Drug Administration (FDA) nor the Malaysian National Pharmaceutical Regulatory Agency (NPRA) has developed comprehensive guidelines for functional cosmetics employing vesicular nanocarriers. Lipidic vesicular formulations are classified as non-biological complex drugs (NBCDs) requiring robust characterisation of primary quality indicators such as particle size, zeta potential, encapsulation efficiency and lipid composition.^150,151^ Additionally, FDA has published a guidance specifically for generic liposomes to enhance research quality, consumer transparency and regulatory rigor.^152^ However, no equivalent framework exists for other lipid-based nano-systems which continue to be investigated and marketed with comparatively fewer regulatory safeguards.^130^

 Furthermore, although formulation and regulatory barriers are being addressed, the clinical evidence for TA-loaded vesicular systems remains insufficient. Most trials are underpowered, with nearly 90% enrolling fewer than 100 participants. The largest study of n = 88 reported MASI score reduction by 48%, but with wide confidence interval (21%-75%) and low statistical power (β = 0.26) limit reliability.^86^ Besides that, follow-up intervals are relatively short ≤ 12 weeks) preventing evaluation of long-term risks such as rebound pigmentation, ethanol-induced SC barrier disruption and rare thromboembolic events.^18,29,99^ Methodological inconsistencies also further undermine validity where outcome measures vary between modified MASI and global 5-point physician scales, while standardised photography and objective tools such as dermoscopy or Wood’s lamp necessary for differentiating between epidermal and dermal forms of melasma are rarely incorporated.^29,105^ Therefore, addressing these gaps will be critical for TA-loaded vesicular systems to progress from experimental formulations to guideline-supported therapies for melasma.

## Conclusion

 Melasma is a chronic and relapsing skin disorder that is multifactorial in origin involving prolonged UVR exposure, genetic susceptibility, hormonal fluctuations and hyperpigmentation triggered by medications or underlying illnesses. Despite advancements in technology and drug development, effective treatment remains challenging. A wide range of interventions including topical formulations, systemic medications and procedural interventions have been explored but many of them demonstrate limited clinical efficacy, high rate of recurrence and undesirable side effects. TA has recently gained recognition as a promising melasma therapeutic option with extensive research conducted on different TA formulations, particularly oral and topical applications. While topical TA alone is considered less effective, it is often preferred due to better patient compliance and lesser side effects. Lipidic vesicular system incorporated with TA represents a promising advancement in melasma treatment, offering enhanced delivery profile, minimized adverse effects and enhanced patient compliance in comparison to conventional methods. However, clinical application remains hindered by several critical challenges: (i) the high cost manufacturing and regulatory complexity, (ii) limited shelf-life and temperature-dependent formulation stability, and (iii) the absence of standardized, validated quality control assays for vesicle characterisation and drug release.

## Future Perspectives

 Moving forward, TA’s incorporation within lipidic vesicles alongside complementary therapeutics such as hyaluronic acid (HA) and microneedle holds potential for optimizing treatment outcomes. These approaches may enhance drug absorption, improve skin hydration and promote overall skin health. Furthermore, innovations in lipidic vesicular nanotechnology could pave the way for personalized dermatological treatments. Future TA formulations may be customized to different types of skin, melasma severity and genetic predispositions, potentially enhancing therapeutic outcomes and patient satisfaction. While significant progress has been made, further research especially multicentred, randomized and dose-ranging clinical trials are needed to optimize standardized protocols, incorporate long-term safety monitoring as well as explore new therapeutic possibilities. Nevertheless, this article underscores the potential of lipidic vesicular formulations for TA to emerge as the first line melasma treatment in the foreseeable future (Supplementary file 1, Table S1). This advancement is contingent upon continued innovation and collaborative efforts within the field to hold the promise of delivering safer, more effective and personalized treatment options to patients.

## Competing Interests

 The authors have no conflicts of interest to declare.

## Ethical Approval

 Not applicable.

## Supplementary Files

Supplementary file 1 contains Table S1.
