# Supplementary material for: Decrypting the Potential of Lipidic Vesicular System for Delivery Enhancement of Tranexamic Acid in Melasma Hyperpigmentation Treatment
Source: Adv Pharm Bull. 2025 Sep 1;15(3):574–87. doi: 10.34172/apb.025.45384 (PMC12703397; doi:10.34172/apb.025.45384)
Supplement: Supplementary file 1 — contains Table S1. [file apb-15-574-s001.pdf]

Adv Pharm Bull. 2025;15(3):574-S1
doi: 10.34172/apb.025.45384
https://apb.tbzmed.ac.ir

Supplementary file 1

Table S1. Different lipid vesicular delivery systems mainly employed for the delivery of tranexamic acid and other hypo-pigmenting agents with their corresponding outcomes.

Vesicular Carrier	Encapsulated bioactive agents	Resulting Outcomes	Reference
Liposomes	Tranexamic acid	- High percentage of drug entrapment (>90%) and increase of physical stability<br>- Reduced erythema index and enhanced moisturizing effects<br>- Significant (>50%) reduced in mMASI scores for melasma as compared to traditional hydroquinone<br>- Enhanced specific drug targeting ability	94-98
Ethosomes/Transethosomes	Tranexamic acid, Linoleic acid, Kojic acid di-palmitate	- Enhanced inhibition of vascularization and angiogenesis as compared to non-liposomal control<br>- Improved drug stability at extreme temperatures<br>- Extended performance on skin lightening and moisturizing capability<br>- Enhanced penetration precisely to the SC depth thereby higher drug release (>95%)	29,111,113-114
Niosomes	N-acetyl glucosamine, Hydroquinone, Kojic acid, Quercetin, Rice bran bioactive compounds, Tranexamic acid	- Enhanced skin penetration as compared to drug's solution form with better depigmentation effects<br>- Increased in drug chemical stability and percentage entrapment efficiency (>90%)<br>- Higher susceptibility to cell toxicity (≥80% viability)<br>- Enhanced clinical effectiveness in hydration, pigmentation, skin elasticity and texture	131-135,138
Transferosomes	Ascorbic palmitate, Niacinamide	- Enhanced 14.1-fold whitening and anti-melasma efficacy<br>- Achieved effective attenuation of oxidative stress and inflammation<br>- Greater drug deposition across the skin SC	140-141
Phytosomes	Cocoa pod extract, Arbutin	- Significant enhancement of antioxidant activity (IC50 of 199.98 ppm)	144-145

		- Higher drug entrapment efficiency comparable to conventional aqueous formulation<br>- Facilitated skin absorption (>80%) and increased efficacy	
